# Discordant circulating fetal DNA and subsequent cytogenetics reveal false negative, placental mosaic, and fetal mosaic cfDNA genotypes

**DOI:** 10.1186/s12967-015-0569-y

**Published:** 2015-08-11

**Authors:** Roger V Lebo, Robert W Novak, Katherine Wolfe, Melonie Michelson, Haynes Robinson, Melissa S Mancuso

**Affiliations:** Department of Pathology and Laboratory Medicine, Akron Children’s Hospital, One Perkins Square, Akron, OH 44308 USA; Maternal Fetal Medicine, Akron Children’s Hospital, One Perkins Square, Akron, OH 44308 USA

**Keywords:** Circulating trophoblast DNA, Discordance, cfDNA

## Abstract

**Background:**

The American College of Obstetrics and Gynecology (ACOG) and Maternal Fetal Medicine (MFM) Societies recommended that *abnormal* cfDNA fetal results should be confirmed by amniocentesis and karyotyping. Our results demonstrate that *normal* cfDNA results inconsistent with high-resolution abnormal ultrasounds should be confirmed by karyotyping following a substantial frequency of incorrect cfDNA results.

**Methods:**

Historical review of our ~4,000 signed prenatal karyotypes found ~24% of reported abnormalities would not have been detected by cfDNA. Akron Children’s Hospital Cytogenetics Laboratory has completed 28 abnormal cfDNA cases among the 112 amniocenteses karyotyped.

**Results:**

Following abnormal cfDNA results our karyotypes confirmed only 60% of the cfDNA results were consistent. Our cases found a normal cfDNA test result followed by a 20 weeks anatomical ultrasound detected a false negative trisomy 18 cfDNA result. One cfDNA result that reported trisomy 21 in the fetus was confirmed by karyotyping which also added an originally undetected balanced reciprocal translocation. Another reported karyotyped case followed by a repeated microarray of pure fetal DNA, together revealed one phenotypically *normal* newborn with a complex mosaic karyotype substantially decreasing the newborn’s eventual reproductive fitness. This second case establishes the importance of karyotyping the placenta and cord or peripheral blood when inconsistent or mosaic results are identified following an abnormal cfDNA result with a normal newborn phenotype without a prenatal karyotype.

**Conclusions:**

*These Maternal Fetal Medicine referrals demonstrate that positive NIPT results identify an increased abnormal karyotypic frequency as well as a substantial proportion of discordant fetal results.* Our results found: (1) a normal NIPT test result followed by a 20 week anatomical ultrasound detected a false negative trisomy 18 NIPT result, (2) a substantial proportion of abnormal NIPT tests identify chromosomal mosaicism that may or may not be confined to the placenta, (3) follow up karyotyping should be completed on the newborn placenta and peripheral blood when the amniocyte karyotype does not confirm the NIPT reported abnormality in order to identify ongoing risk of developing mosaic symptoms, and (4) karyotyping all high risk fetuses tested by amniocentesis defines the 24% of chromosome abnormalities not currently screened by NIPT.

**Electronic supplementary material:**

The online version of this article (doi:10.1186/s12967-015-0569-y) contains supplementary material, which is available to authorized users.

## Background

Interest in circulating cell free DNA fragments began when transplacental transmission of fetal cells into the maternal circulation was demonstrated by detection of fetal erythrocytes [1]. Subsequently, fetal lymphocytes were also found in maternal blood [2]. Then a fluorescence activated cell sorter (FACS) isolated whole fetal cells from maternal circulation [3] including circulating Y chromosome carrying fetal cells from a woman who conceived a male fetus eight (8) years earlier [4]. Nevertheless, DNA analysis of microdissected intact circulating fetal cells identified with a specific fetal antibody correctly diagnosed five (5) of five (5) fetuses at-risk for three (3) different genetic diseases [5].

Other investigators reported the majority of circulating fetal DNA (cfDNA) in maternal plasma is derived primarily from the trophoblast [6, 7]^a^. This was selected as a preferred source of fetal DNA to be tested because it has an average half-life of 16.3 min post-delivery so that levels are undetectable within hours post-partum [8, 9]. This cfDNA can be detected reliably in the maternal circulation by 7 weeks gestation and its relative proportion of total circulating DNA increases with gestational age [7]. Subsequently, a few selected chromosome-specific sites were tagged along with control sites, all tags sequenced millions of times, and the total frequencies compared to normal DNAs to identify whole chromosome aneuploidy in the admixed fetal DNA: trisomy when increased and monosomy when decreased.

Testing cfDNA is being applied clinically as a noninvasive fetal screening test to identify the most common chromosome aneuploidies [10, 11]. Following reports of inconsistencies between circulating fetal DNA analysis and subsequent karyotypes, the joint American College of Obstetrics and Gynecology (ACOG) and The Society of Maternal-Fetal Medicine **(**SMFM**)** Committee Opinion stated, “A negative cell free fetal DNA test result does not ensure an unaffected pregnancy. A patient with a positive test result should be referred for genetic counseling and should be offered invasive prenatal diagnosis for confirmation of test results” [12].

Bianchi and collaborators have completed multicenter studies reporting fetal placental DNA in maternal circulation to be a substantially improved indicator of fetal aneuploidy over prior screening [10, 11]. The overall negative predictive value was 100% (95% confidence interval [CI], 99.8–100). The positive predictive value for *trisomy 21* was *45.5%* (95% CI, 16.7–76.6) and for *trisomy 18*, the positive predictive value was *40.0%* (95% CI, 5.3–85.3) [11]. These positive predictive values are consistent with our Maternal Fetal Medicine specialists’ submitted samples (see “Results”).

Placental mosaicism has been reported in 0.8 to >2% of viable fetuses studied by chorionic villus sampling at 10–12 weeks gestation with a cytogenetic abnormality in the placenta [6, 13–18]. In one study of 11,200 cases¸ available follow up found 20% of placental mosaic cases were also in fetal tissues [14]. This same study confirmed rates for fetal mosaicism between 7.6% for autosomal trisomy to 77.8% for a marker chromosome [14]. Intrauterine growth restriction (IUGR) and small for gestational age (SGA) infants in about 10% of pregnancies are both associated with an increased risk for perinatal morbidity and mortality. Chromosomal mosaicism confined to extra embryonic tissues (CPM) has been observed in over 20% of pregnancies with idiopathic IUGR [6, 7].

Discordant placental DNA in maternal circulation, ultrasound, and karyotypes in this study emphasize the importance of invasive fetal testing and targeted follow up analysis of term placenta and newborn blood. Of the six cases with discordant cfDNA results, one case with an abnormal fetal ultrasound following a normal reported circulating placental DNA in maternal blood revealed trisomy 18 in all fetal amniocytes (Table 1, case 1). Another four cases had discordant trisomy 21, monosomy X, and trisomy 18 results (Table 1, cases 2–5). A molecularly balanced translocation detected by follow up karyotyping a trisomy 21 fetal cfDNA result (Table 1, case 6) reflects the limitations of testing only the most frequent chromosome abnormalities by NIPT. Together these cases emphasize the importance of counseling and confirmation of inconsistent circulating placental DNA and ultrasound results by ongoing testing in amniocytes, placenta and/or newborn blood.

**Table 1:** Six listed discordant NIPT results (case numbers 1–6) among the nine confirmed abnormal NIPT results (case numbers 7–15)

Case number	NIPT	Karyotype	Phenotype
1	2 × 21,13,18, X MaterniT21 Specificity: 99.6% [CI 99.2–99.6% for chromosome 18]	Amniocentesis 47,XX, +18	Affected newborn Expired postpartum
2	Trisomy 18 [9W3D] MaterniT21 Sensitivity: >99% Repeat normal [22W]	Term Placenta: 46,XY	Normal newborn:
3	Trisomy 21 >99% harmony	Amniocentesis: 46,XY[18]/Fish[50]	Normal newborn Blood: 46,XY
4	Trisomy 21 validated at 100%	Amniocentesis: 46,XY[28]	Placenta: 47,XY, +21[18]/46,XY[2] Newborn Blood: 46,XY
5	Monosomy X Ariosa 99%		Newborn blood: 45,X[1]/46,X,dup(X)(q13q21.3)[49]
6	Trisomy 21	Amniocentesis: 47,XY,t(5;9)(p15;q12)+21	
	Microarray: trisomy 21		
	Balanced t(5;9) not detected		
7–11	Trisomy 18	47,X_, +18	
12–15	Trisomy 21	47,X_, +12	

## Methods

Prior to offering one of the circulating fetal DNA screening tests to pregnant mothers, patients referred to our regional Maternal Fetal Medicine specialists are counseled that these NIPT tests for circulating placental DNA in maternal circulation are screening tests and as such are not entirely diagnostic. Of the four testing laboratories that originally analyzed fetal chromosome aneuploidy in cfDNA, maternal blood samples were sent to either: (1) Sequenom testing with MaterniT21, or (2) Ariosa Diagnostics testing with Harmony at Integrated Genetics (Table 1^b^). Following a positive screening test result, amniocentesis was offered to all the cases with an abnormal fetal result in circulating placental DNA test result according to our initial protocol and confirmed by the ACOG recommended protocol [12]. Given a gestational age of 27 weeks of a fetus after testing positive for trisomy 18 by NIPT, this single fetal case was retested by a second NIPT test that gave a normal result.

FISH analysis of direct amniocytes was completed on fixed nuclei hybridized to probes for chromosomes 13, 18, 21, X, and Y according to standard Vysis protocols (Abbott). Karyotyping was completed on amniocytes, placental biopsies, and/or newborn blood samples by tissue culture, trypsin digestion and cell harvesting, hypotonic swelling, fixing, and slide preparation according to the standard protocols. Metaphase karyotypes were banded by trypsin digestion followed by Wright’s staining and G-banded analysis [Akron Children’s Hospital Cytogenetic Laboratory].

Term placentas from positive testing fetuses were placed into medium, stored cold, and transported at 4°C prior to analysis. Pathologists Dr. Kimberly Eickholt or Dr. Dimitris Agamanolis obtained biopsied samples of villous rich tissue from each placenta. According to our placental Products of Conception (POC) protocol, cytogenetic technologists further dissected the chorionic villi from the maternal deciduas, disbursed the cells with collagenase, and seeded the cells in two independent flasks. The dissected chorionic villous sampled (CVS) cells that grew in two flasks were karyotyped. In addition, an aliquot of collagen digested villous cells were prepared in hypotonic and fixed for possible additional FISH analysis. Chorionic villous cells that grew in culture were karyotyped according to standard protocols.

The placenta from the circulating placental DNA testing positive for Turner Syndrome (45,X) (Table 1, case 5) was biopsied in four places and tested as two composite samples, paraffin embedded, and the cells removed as a core, deparaffinized, digested overnight in collagenase to disburse the cells, the cells fixed, and attached to the surface of a microscope slide. FISH analysis proceeded by hybridization to chromosome X labeled with spectrum green, Y labeled with spectrum orange, and 18 labeled with spectrum aqua (Vysis) which were scored and captured independently for each nucleus.

Akron Children’s Hospital Internal Review Board Chairman Dr. Robert Novak discussed and assured compliance with required clinical and research applications of these results at each step in the process. The patients’ results reported in detail have a signed permission to publish these data.

## Results

### Amniocentesis summary beginning with first discordant NIPT result

One hundred three (103) amniocentesis samples (Table 2) have been karyotyped by our Akron Children’s Hospital Cytogenetics Laboratory beginning with the first sample for which the karyotype was inconsistent with the NIPT result (NIPT-noninvasive prenatal testing; cfDNA-circulating fetal DNA). Of these fetuses, 53 were sonographically abnormal (Table 2). Fifteen (15) of 97 were abnormal by NIPT including 9 confirmed by karyotyping, while the remaining 6 had false positive, inconsistent, or mosaic results and one (1) with an abnormal ultrasound had a false negative trisomy 18 result by NIPT (six selected cases listed in Table 1). This experience includes ~60% confirmed abnormal NIPT fetal test results. These confirmed results are consistent with the positive predictive test value of 40% for trisomy 18 reported for a very large tested series of pregnant patients (Bianchi et al. 2014). The correctly identified chromosome abnormalities include five trisomy 18 fetuses and four trisomy 21 fetuses. The discordant NIPT results involve trisomy 18, trisomy 21, or monosomy X (Table 1). No fetus was reported to have originated from in vitro fertilization.

**Table 2:** Additional indications for all karyotyped amniocenteses

Total number	Indication
53	Abnormal ultrasounds: Including heart defects (14); IUGR (4); echogenic bowel (4) spina bifida (3), skeletal abnormalities (2) choriod plexus cyst oligohydramnios; cystic hygroma
26	Increased marker screening risk: 2 atypical abnormal karyotypes
12	Advanced maternal age
9	Family history: genetic disease including metabolic, chromosomal, Rh
2	Other
1	Parvovirus exposure: 46,XY karyotype

Completed while testing the NIPT cases (Table 1).

### Trisomy 18 fetal results

**Fig. 1:**
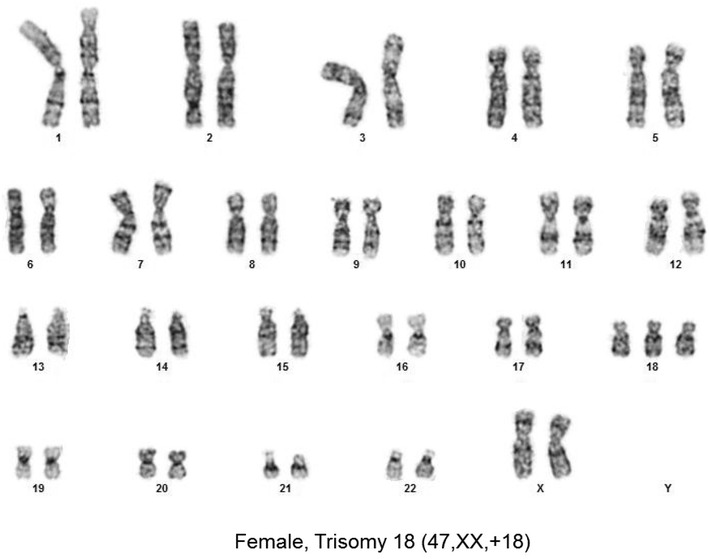
Trisomy 18 fetal amniocytes from a fetus normal by NIPT.

Five NIPT results reflecting fetal trisomy 18 were confirmed by follow up karyotypes of amniocytes. In contrast, one fetus was reported in an early test to be normal for all chromosomes tested by MaterniT21 including chromosome 18 with a specificity of 99.6% and a CI 99.2–99.6% for chromosome 18 (Table 1, case 1). The Maternal Fetal Medicine specialist followed this result with an ultrasound that found multiple abnormalities including left sided diaphragmatic hernia with herniation of bowel and liver, echogenic kidneys, and bilateral hydronephrosis. When amniocentesis was offered and completed based upon an abnormal ultrasound, a trisomy 18 fetal karyotype was found in all 28 of 28 amniocytes examined (Fig. 1). More than half of our Ohio patients elect to carry a trisomy 18 fetus. Intensive care units at 24 immediately surrounding hospitals in our area have adopted a protocol to only provide comfort care to trisomy 18 fetuses surviving delivery to avoid protracted ICU stays. Given this information about ICU protocols, this mother elected to continue the pregnancy, delivered at her local hospital, and then held the newborn for the 40 min it survived after birth. This case emphasizes the importance of further testing to optimize ongoing care when an abnormal fetal ultrasound is observed following a normal NIPT result.

**Fig. 2:**
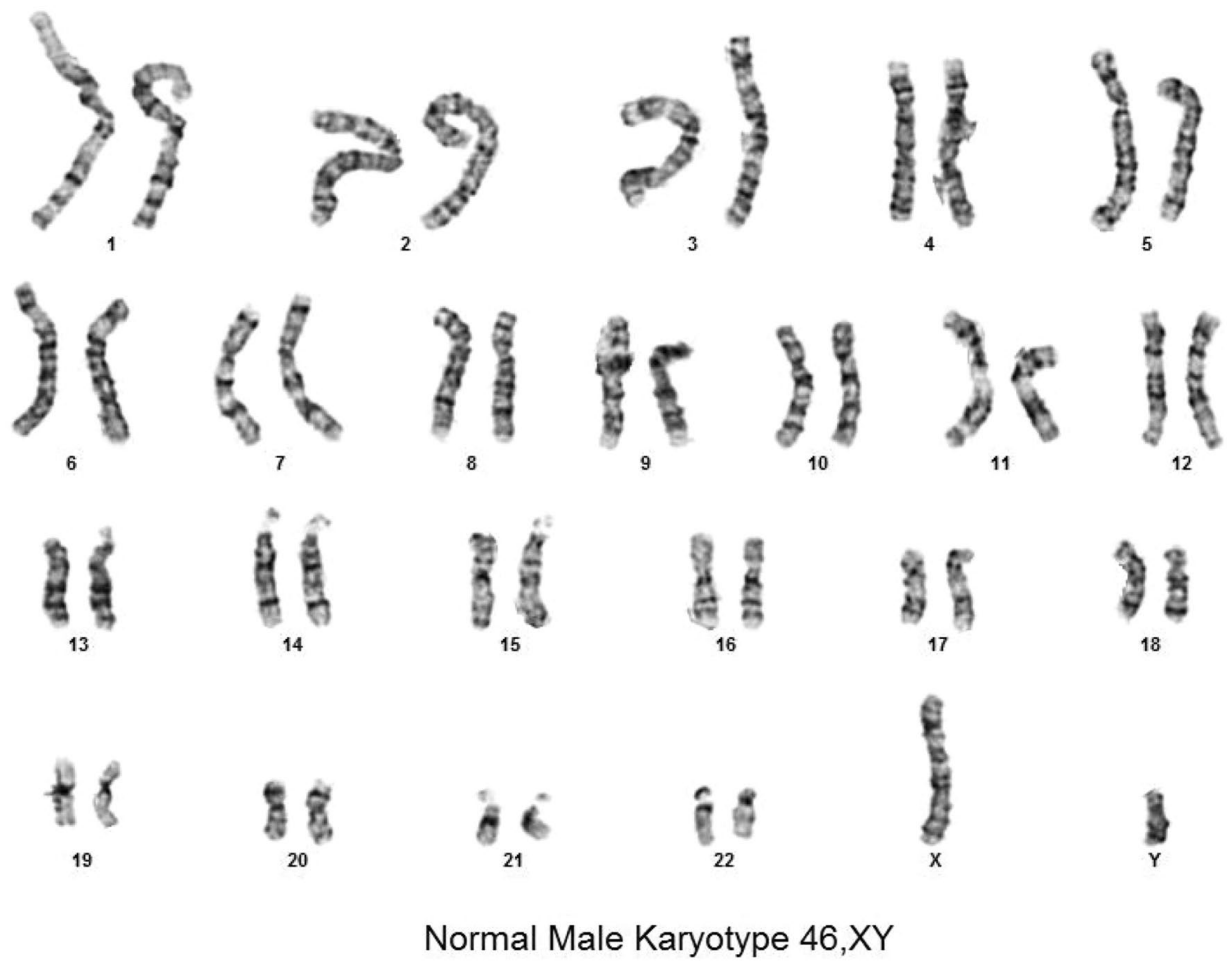
Normal male term placental karyotype. Trisomy 18 by NIPT.

Another patient was reported to be carrying a 9 week 3 day gestation fetus “Positive for Trisomy 18” with a sensitivity of 99.9% and a confidence interval of 92.4–100% by MaterniT21 (Table 1, case 2). This report continued, “DNA test results do not provide a definitive genetic risk in all individuals… (and) does not replace the accuracy and precision of prenatal diagnosis… (This patient) should be referred for genetic counseling and offered invasive prenatal diagnosis for confirmation of test results”. The obstetrician ordered the NIPT test repeated at 23 weeks gestation and this result was reported to be “normal for chromosome 21, 18, and 13 material” with a specificity of 99.6% and a confidence of 99.2–99.8%.” The lab director wrote, “Repeat testing was negative for trisomy 18 which was originally reported as positive… Sample identity testing confirmed that original and redrawn sample were from the same individual. A biological interference is suspected but this discrepancy remains unresolved”. The patient elected to continue the pregnancy and delivered a *healthy, normal male*. Four biopsies were obtained from the “Mature placenta with patchy mild villous edema and chorangiosis”. Karyotyping biopsies from 4 biopsies of dissected chorionic villi grown in two cultures found 30 of 30 cells with normal male karyotypes (Fig. 2).

### Trisomy 21 fetal results

Four (4) NIPT results indicating each fetus had trisomy 21 were confirmed by follow up amniocyte karyotypes. The fifth patient was reported to be carrying a 16 week 5 day fetus positive for Trisomy 21 by Ariosa (Table 1, case 3). Subsequent amniocentesis found 66 of 66 metaphase cells with a normal two copies of chromosome 21 by both FISH and karyotyping. The newborn phenotype was normal (Table 1, case 3).

Another patient (Table 1, case 4) reported herein tested positive for trisomy 21 by circulating placental DNA with the report stating the likelihood of this trisomy 21 result being correct was “validated at 100%” [19; Ariosa Diagnostics]. According to ACOG Committee Guidelines, all cases of circulating placental DNA (cfDNA) that have tested positive for a fetal chromosome abnormality are counseled that the positive result was obtained by a screening test and that no irreversible decision should be made prior to completing a subsequent amniocentesis. According to the recommendations of ACOG, Ariosa, and Akron Children’s Maternal Fetal Medicine Division, a follow up amniocentesis was offered during counseling. The mother elected to complete amniocentesis that found 90% of 50 uncultured interphase amniocyte nuclei with normal chromosome 13 and 21 patterns and no nuclei with a trisomy 21 pattern. Subsequent karyotypes of cultured cells found 28 of 28 amniocytes with normal 46,XY male karyotypes. A follow up Level II ultrasound found the fetus did not have any visualized phenotypic abnormalities or soft markers of aneuploidy. The patient elected to continue the pregnancy.

**Fig. 3:**
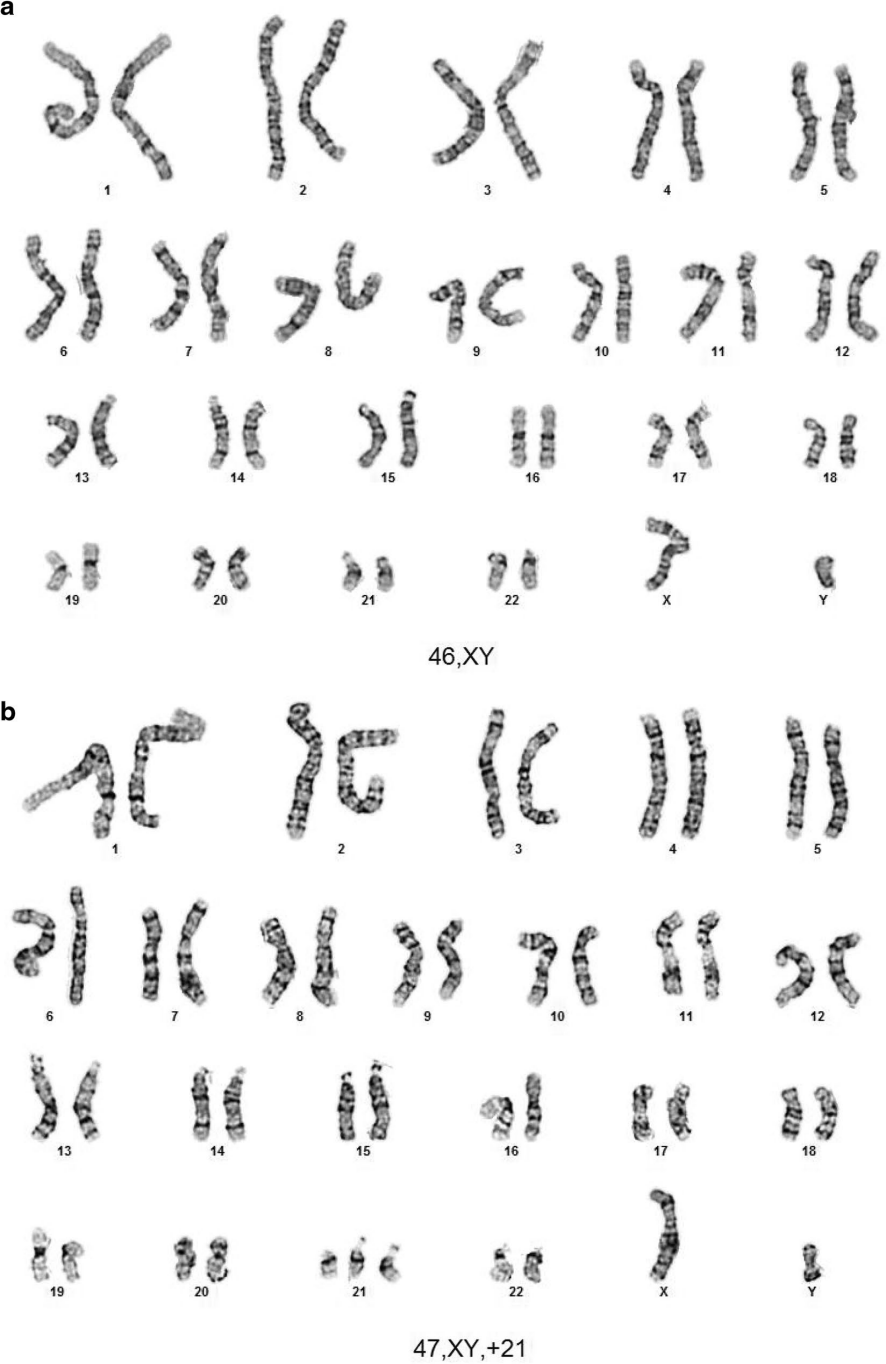
**a** Peripheral blood from a newborn with trisomy 21 by NIPT. **b** Placenta from newborn reported as trisomy 21 by NIPT.

**Fig. 4:**
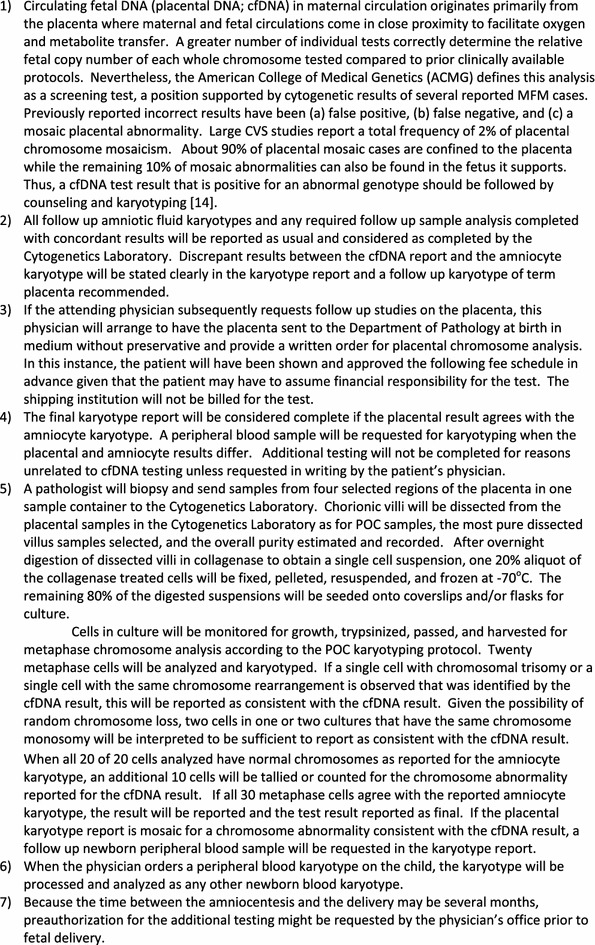
Protocol for further fetal testing following a report of abnormal placental DNA in maternal circulation.

Because placental mosaicism was previously detected in circulating placental DNA [14] prior to delivery, this fetus’ term placenta was requested along with a newborn blood sample. This G4P2 fetus was subsequently delivered by C-section at 38 weeks gestation with symmetric growth restriction but with no trisomy 21 associated phenotype. The requested fresh placenta stored cold in culture medium was transported to our cytogenetics laboratory for follow up analysis according to our standard POC protocol (Figs. 3, 4). A pathologist biopsied the placenta in four locations and the biopsied chorionic villi were further dissected to an estimated 95% purity, digested in collagenase, and cultured. CVS karyotypes from both of the two successfully cultured biopsies included eighteen (18) 47,XY, +21 cells and two (2) 46,XY cells^c^. These results define the origin of the abnormal trisomy 21 circulating fetal DNA result and explain the newborn’s symmetric growth restriction.

Given ~20% of reported placental mosaicism is also found in the fetus [14], a newborn blood sample from this 6  day old was requested and karyotyped as a second tissue sample. All 20 of 20 cells were normal 46,XY male. Together with the original amniocyte karyotype of 46,XY normal male in 28 of 28 amniocytes, these results support the interpretation that the 47,XY, +21 cells were confined to the placenta^c^ emphasizing the importance of karyotyping amniocytes and newborn cord or peripheral blood lymphocytes in phenotypically normal newborns. Together all these results support the interpretation that the abnormal trisomy 21 karyotype was confined to the placenta and the fetus has a normal constitutional karyotype (Fig. 3). Following this counseling and testing saga, this patient expressed her gratitude for the thorough counseling and testing she had received throughout her pregnancy, emphasizing the importance of supportive patient care.

A mosaic trisomy 21/tetrasomy 21/normal karyotype confined to the placenta was reported was found in a CVS sample in the 1990s that may have been related to the tetrasomy 21. Other mosaic placentally confined chromosome aneuploidies were reported in 0.8 to >2% of viable fetuses. Taken together, these results are consistent with previously reported confined placental mosaicism associated with IUGR [13–15]. The biopsied placental karyotypes also explain the results of Ariosa’s cfDNA test indicating a fetal trisomy 21 genotype and further emphasize the importance of an ACOG recommended follow up karyotype on amniocytes [12] plus our newborn blood karyotype to determine whether both these tissues have entirely normal chromosomes. Standard laboratory protocols for POC analysis that culture dissected chorionic villi stored at 4°C up to 5 days typically result in a fetal karyotype >90% of the time while placental villi kept at room temperature have also remained viable for 3 days. Subsequent FISH analysis of paraffin embedded nuclei added by Pathologists identified the common chromosome aneuploidies in most of the remaining cases that failed to grow in culture [20, 21]. Our laboratory protocol accompanying this report (Figs. 3, 4) could be adopted readily into the College of American Pathologists (CAP) guidelines.

### Monosomy X results

Case 5 (Table 1) is a fetus that presented at 27 weeks 6 days with intrauterine growth retardation. Given the fetus’ late gestational age, NIPT was selected as the most appropriate test and a maternal blood sample was submitted to Ariosa. The 27.3% of circulating fetal DNA indicated the fetal karyotype was monosomy X with a probability greater than 99/100 (99%). The patient carried the fetus. At birth the fetus was small for gestational age with a sacral dimple, jaundice, and poor feeding that resolved but was otherwise phenotypically normal. The 6 days infant was discharged home. A thorough physical exam by a geneticist for 30 characteristics at 2 months and again at 3 months revealed no discernible abnormalities.

**Fig. 5:**
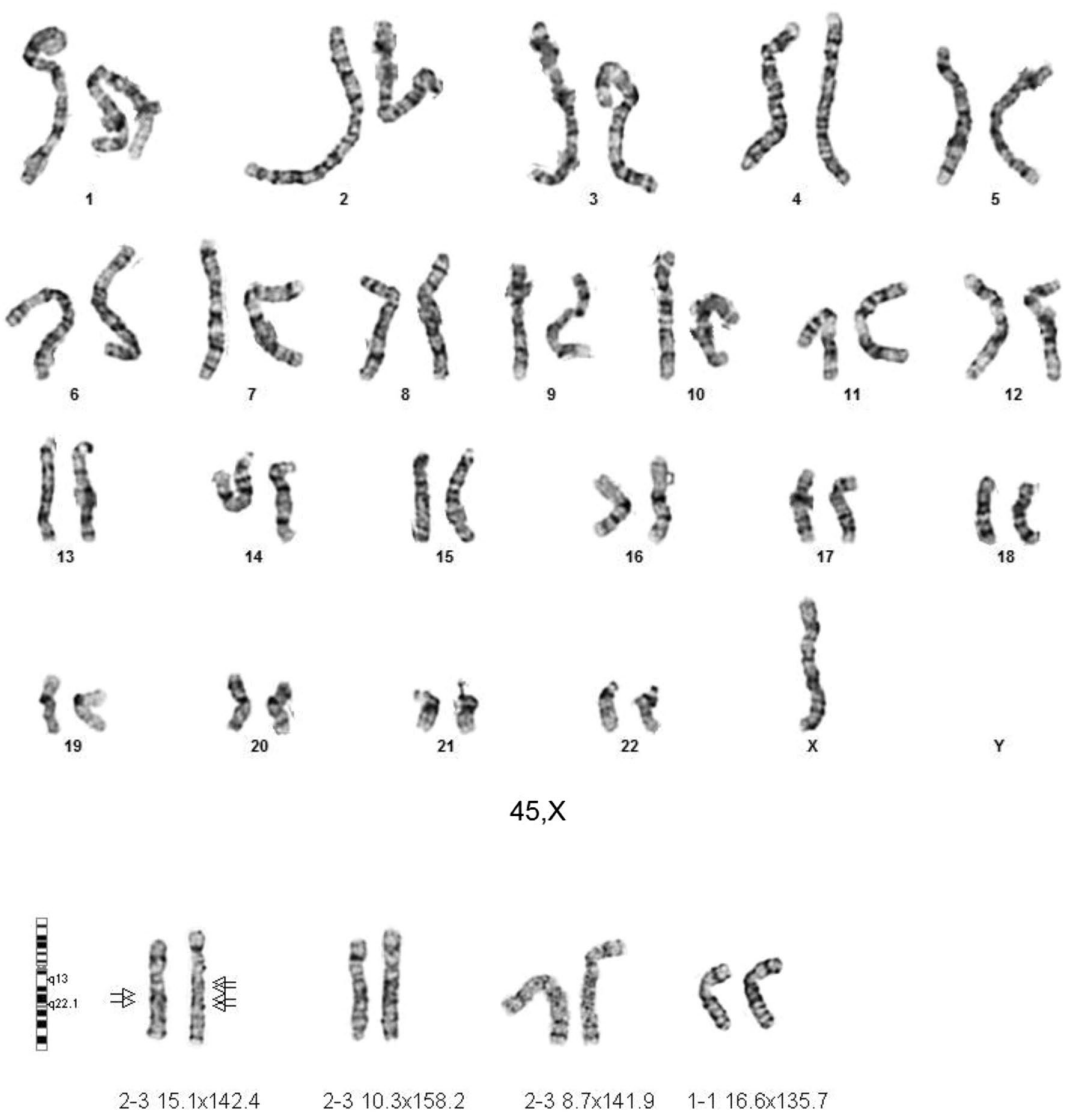
GTW-banded karyotypes of monosomy X and Xq duplication on patient 3. The third case had a mosaic karyotype with Monosomy X in 9 cells and an X chromosome with a duplication of the long arm 45,X[9]/46,X,dup(X)(q13q22.1) [41]. The G-banded X chromosomes in 9 pf 50 cells are shown for the 45,X cell line (*top*), and 4 cells of the second cell line with the duplicated chromosome regions indicated on the normal idiogram to the left (*bottom*). The normal X chromosome is illustrated for five metaphase cells to the left of the X chromosome with the long arm duplication to the right of it. The *arrows* are drawn to the Xq21.1 and Xq21.2 bands. [Illustration courtesy of James Malone, Supervisor, Akron Children’s Cytogenetic Laboratory].

**Fig. 6:**
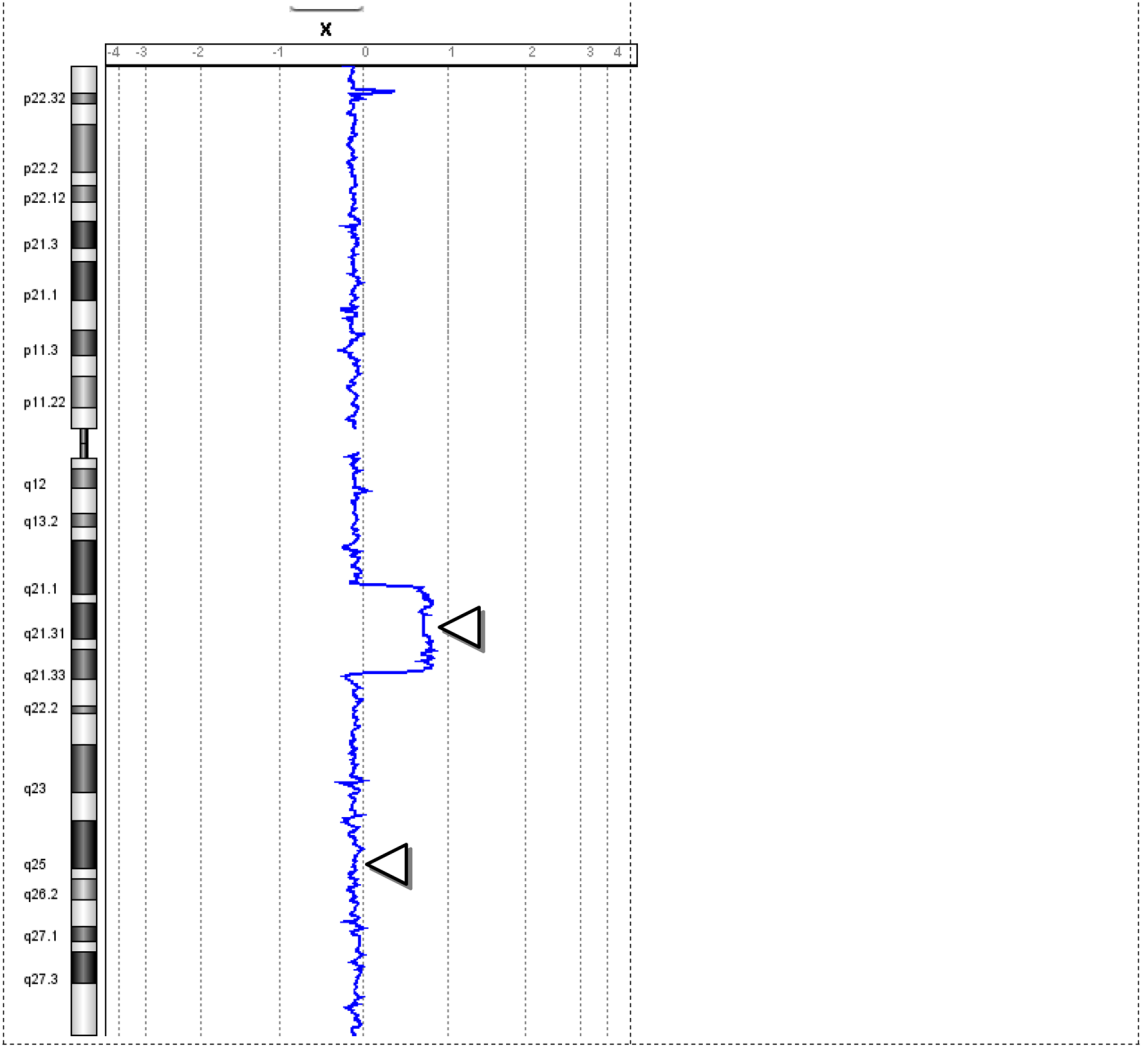
X chromosome microarray result on patient 3. The primary result from top to bottom is two copies of the X chromosome in ~120 Mb of the X chromosome in ~85–90% of the cells and one copy of the X chromosome [45,X] in ~18% of the cells (*lower triangle*). One extra copy of the X chromosome region [46,X,dup(X)(q21.1q21.3)] in ~82% of cells (upper triangle). FISH with a duplicated region confirmed that the cells with a single X chromosome contained only the normal X and each X chromosome had a single XIST gene in band Xq13. The *horizontal axis* is drawn and the result plotted as the natural logarithm of the data to ensure quantification of multiple copies in the same cell occasionally seen as amplified copies. The image is provided by Daniel Pineda-Alvarez M.D., FACMG, Associate Microarray Director, GeneDx. The difference in the GTW banded chromosome breakpoints is based upon lower resolution FISH analysis using a limited number of chromosome probes and measuring the distance on idiograms that are not based upon measured band location.

Karyotypes and FISH analysis of newborn blood found 45,X[9]/46,X,dup(X)(q13q21.3)[41] (Fig. 5) with a single X inactive specific transcript (XIST) gene locus in band Xq13 by FISH. Microarray analysis confirmed the newborn’s 45,X mosaic cell line in a small fraction of the cells and a second more frequent cell line with a duplication on the X chromosome long arm dup(X)(q21.1q21.33) spanning from 83,317,750 to >96,924,899 bp (Fig. 6). This duplication resulted in 3 total chromosome X long arm region copies given 1 copy on the normal X chromosome and two copies on the X with the duplication. The microarray X chromosome plot (Fig. 6) also illustrates that cells with 45,X comprise ~15 to >20% of the total number of cells in this independent sample. These mosaic 45, X cells explain the monosomy X result in a portion of placental DNA tested in maternal blood which is consistent with IUGR at 27 weeks. FISH analysis of the parents’ blood had normal patterns indicating the newborn’s chromosome abnormalities are de novo.

**Fig. 7:**
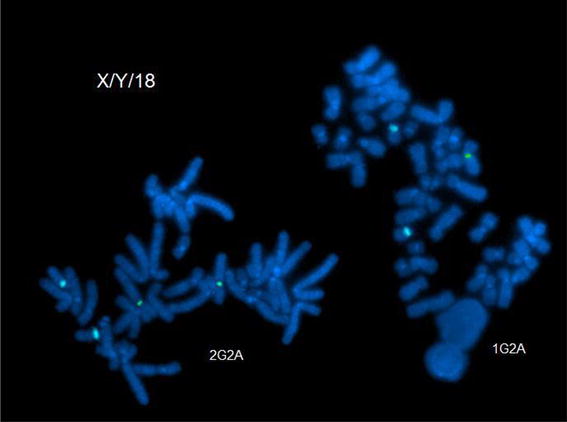
From Fetal Case 5. *Left* DAPI stained karyotypes of normal and Xq duplication chromosome centromeres labeled *green* and with control chromosome 18 centromere labeled *aqua*.

**Fig. 8:**
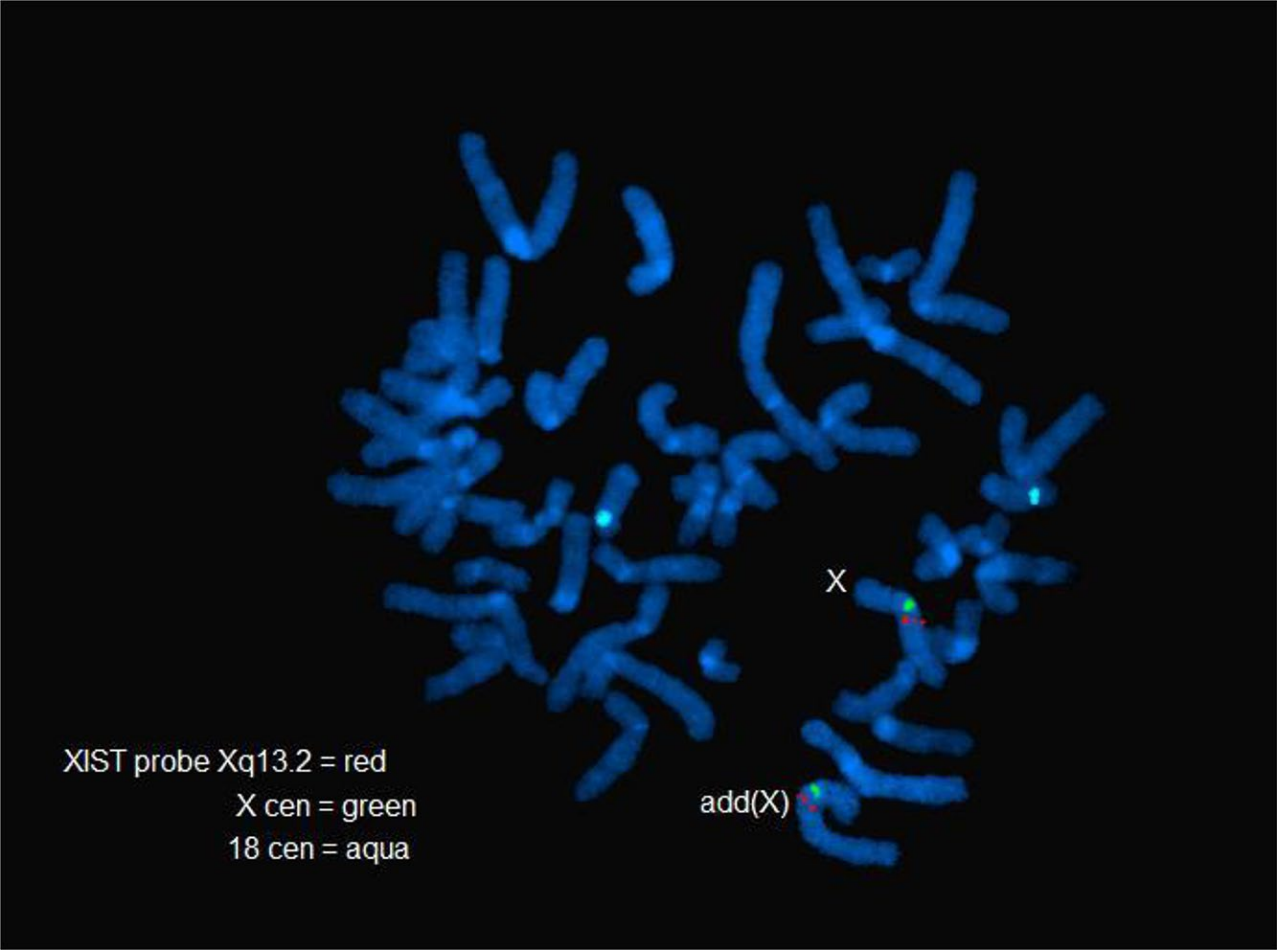
Case 5. Fetal DAPI banded karyotypes of normal and Xq duplication [add(X)] chromosome centromeres labeled *green* and the control chromosome 18 centromere labeled *aqua*.

Uniparental disomy was also identified by polymorphic microarray analysis in this newborn’s DNA from Xq21.33 to Xq28. This is most likely explained by unequal X long arm recombination in parental Meiosis I that resulted in a duplication followed by cosegregation of the normal and duplicated X chromatids to the same gamete. Given no clearly defined paternal or maternal imprinted gene regions on the X chromosome, this finding below the duplicated chromosome region on dup(X)(q21.1q21.3) does not modify patient prognosis. Given a single XIST gene locus on both the patient’s normal and derivative X chromosomes without an extra XIST copy by FISH, this copy number minimally modify a female newborn’s phenotype provided the duplication breakpoints did not result in an X-linked dominant genetic disease. This is in contrast to an X chromosome with two XIST genes that typically inactivate nearly all X chromosome genes to mimic a Turner phenotype [22]. However, this patient’s long term prognosis is significantly modified when she reaches reproductive age with a substantially increased risk of chromosomally abnormal gametes derived from the duplicated chromosome region resulting in a substantially increased risk of miscarriage. This case emphasizes the importance of karyotyping a phenotypically normal appearing newborn after it tests abnormal by NIPT (Figs. 7, 8).

### Trisomy 21 and a balanced reciprocal translocation

**Fig. 9:**
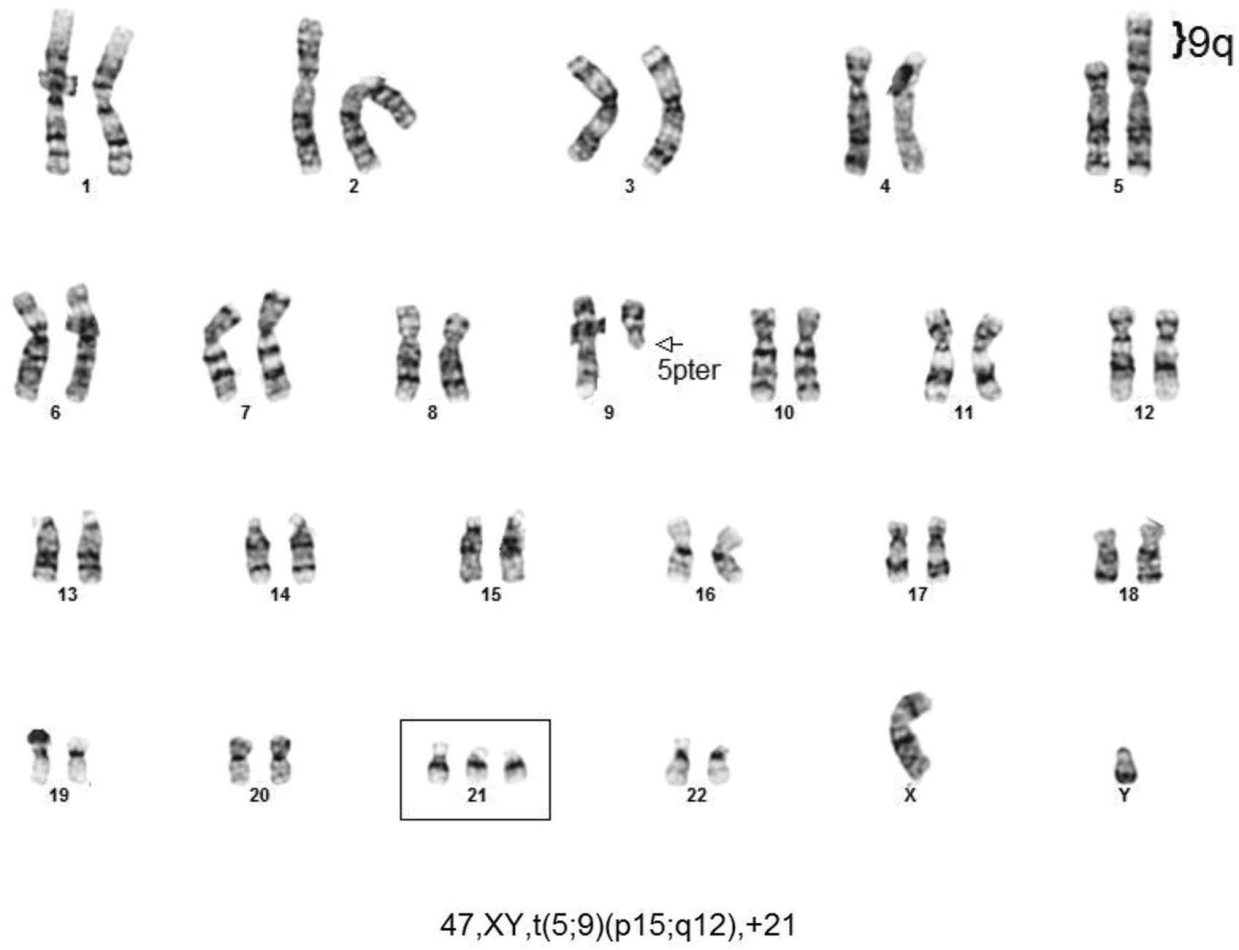
Case 6 karyotype.

**Fig. 10:**
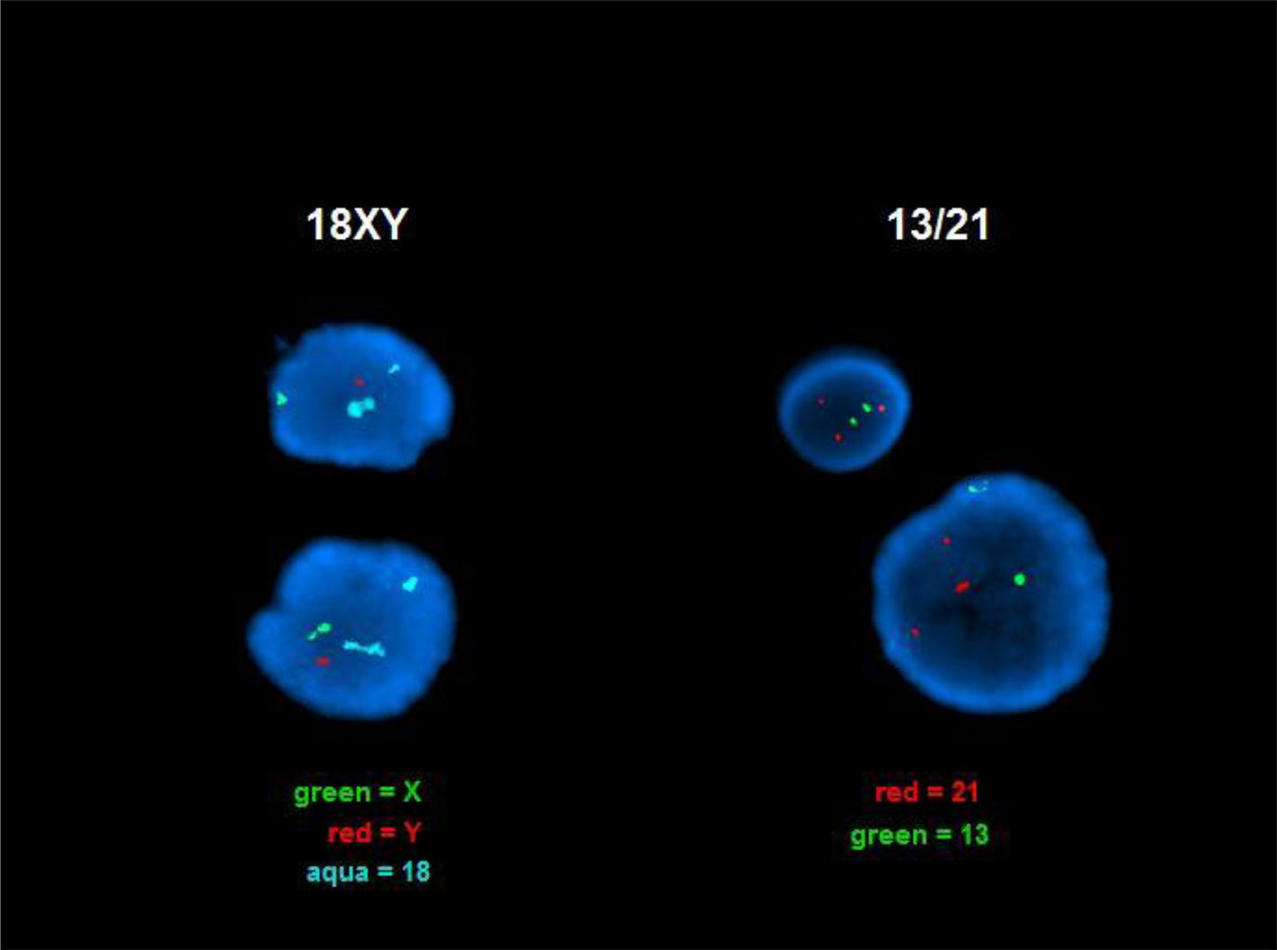
Case 6. *Right* DAPI stained interphase nuclei with three chromosome 21 centromeres labeled in* red* and* green* and two chromosome 21 centromeres labeled with control chromosome 13 centromere probe labeled in *green*. This confirms that three copies of the Down Syndrome critical region are found in each nucleus and therefore the above karyotype will result in Down Syndrome. *Left* The normal Y chromosome centromere is labeled in *red*, the normal X chromosome centromere in *green*, and to different since normal chromosome 18 centromeres labeled in *aqua* reflect a normally variable difference in the number of centromeric repeats.

One of five trisomy 21 patients tested by NIPT and confirmed to have trisomy 21 also had a balanced reciprocal translocation characterized by G-banded karyotyping: 46, XY, t(5;10)(p15;q12). (Table 1, case 6; Figs. 9, 10) A follow up microarray was completed that confirmed the translocation is submicroscopically balanced because a submicroscopic deletion or duplication at the translocation breakpoint could not be detected and would not have been detected by a microarray alone. This specific case emphasizes that following abnormal NIPT results by karyotyping also detects any tested additional chromosome abnormalities not currently screened by NIPT.

## Discussion

These cases illustrate the following principles: (1) Noninvasive prenatal testing is enhanced by thorough ongoing patient counseling. (2) Abnormal ultrasounds with a recognizable genetic etiology should be offered an invasive procedure to test for a genetic abnormality even when placental DNA screening was normal. (3) The reported frequencies of abnormal circulating placental DNAs is lower than the reported ~1.5% mosaic CVS frequency, suggesting that many chromosomally mosaic placentas are not detected by NIPT. (4) Given that ~20% of reported placental mosaicism is also detectable in the fetus [14], amniocyte karyotypes should follow any prior abnormal circulating placental DNA result and karyotyping newborn cord or peripheral blood should be considered when the amniocyte karyotype and/or newborn phenotype is normal [5]. Because circulating placental DNA is more accurate than previous fetal screening tests in experienced laboratories, the frequencies of abnormal NIPT results have increased ~10-fold when selected high risk patients are referred to more specialized Obstetricians and Maternal Fetal Medicine Clinics.

### Ultrasounds are an important screening adjunct

Our initial reported karyotyped case demonstrated that fetuses with abnormal ultrasounds and a recognizable etiology should be offered an invasive diagnostic procedure to test for the most likely genetic origin of the phenotype. Improved ultrasound devices, training, and experience enable obstetricians to characterize abnormal and normal fetal phenotypes in spite of an erroneous test result. Thus judiciously interpreted ultrasounds provide an important check to any laboratory test result.

### Enrichment of inconsistent placental results in reference centers

Placental mosaicism has an overall frequency of ~1.5% in five large chorionic villus sample studies [6, 13–17]. Among these mosaic cases that may be detected by testing circulating placental DNA, ~80% are confined to the placenta while ~20% are also found in the fetus. Because a normal amniocentesis karyotype following an abnormal circulating placental DNA result only tests one fetal tissue source, additional assurance that this result reflects the fetal status could be obtained by completing a 20 weeks anatomic ultrasound for phenotypic abnormality and IUGR. Among our 103 amniocenteses completed with 15 previously tested by NIPT, 9 of 15 were confirmed to have abnormal karyotypes and 7 of 15 had discordant karyotypes reflecting a substantial improvement over prior tests. Testing polymorphic sites that regularly differ in paternal DNA polymorphisms is anticipated to further increase NIPT reliability.

One of our trisomy 21 NIPT results (Table 1, case 4) followed by a normal fetal karyotype and a mosaic trisomy 21 placenta, indicates that large future studies that include fetal placental analysis when discrepant results are found will determine the true frequency of discordant NIPT results. Furthermore, the reported frequency of 0.1% false positive results in all risk populations by experienced laboratories [9] predicts that the 1.5% of mosaic fetal placentas have not all been detected failing to characterize some cases of IUGR.

### Future approaches

**Fig. 11:**
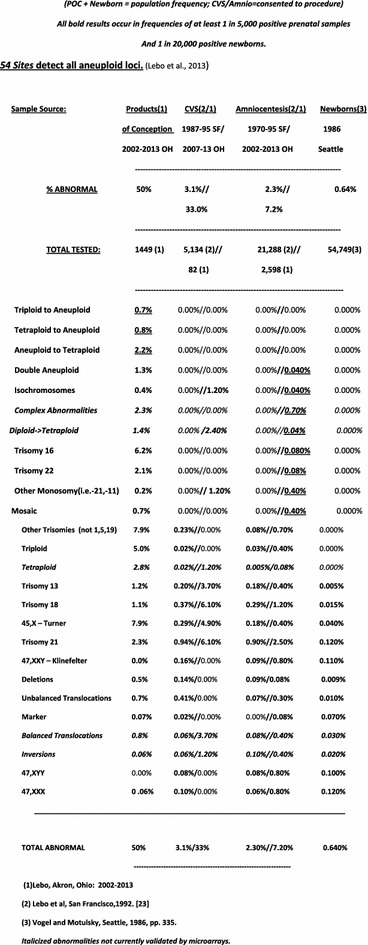
26 Abnormal karyotypic categories detected at 54 sites listed by decreasing severity. (Reprinted from Lebo and Tonk [26]).

A subsequent more informative fetal screening test with substantially increased reliability based upon the many tested sites at each targeted chromosome region would be completed by adding the 54 sites that would have detected all unbalanced fetal chromosome rearrangements among our 938 abnormal karyotypes (Fig. 11; [21, 23]). In contrast, the 2,500,000 genomewide microarrays [21] would not have detected any reported additional cases in the most recent 12 years [11, 24]. Even when smaller targeted aneuploid sites are tested, the genomic region will have many informative polymorphic sites to query to meet the current standards applied to whole chromosome testing. Another goal would screen de novo submicroscopic chromosomal aneuploidies in placental DNA [11, 25, 26]. The protocol requiring follow up analysis of all abnormal results will be prudent for all ongoing and pending karyotype applications.

### Thorough analysis optimizes patient care

Rapid DNA testing has enabled new test applications at an unprecedented rate. Reliably predicting all the consequences of each application is not always possible. Nevertheless, given the sophisticated data presented by these enabling technologies, a thorough understanding of all existing reported data assists in optimizing ongoing interpretation and intervention. Ongoing patient counseling optimizes outcomes. ACOG supports a recommendation to compile and report all inconsistent results between abnormal circulating fetal DNA and normal fetal cells. Our results further emphasize the importance of (1) completing invasive prenatal diagnosis of all abnormal ultrasounds in the face of existing normal placental DNA results, and (2) completing results on newborn bloods to either confirm a normal invasive prenatal karyotype is not mosaic in another tissue or to test the newborn karyotype when a prenatal karyotype was not obtained following an abnormal ultrasound.

## Conclusion

This manuscript (1) reports and explains the predicted increased frequency of discordant placental DNA results to fetal karyotypes at Maternal Fetal Medicine referral centers, (2) demonstrates the importance of follow up ultrasound with normal NIPT results, (3) karyotyping phenotypically normal newborns with abnormal NIPT results, (4) ongoing thorough counseling, and (5) provides our laboratory protocol for follow up analysis of placenta along with cord or peripheral newborn blood (Additional file 1).

Our 103 amniocenteses completed for all indications since the first case of inconsistent results between circulating trophoblastic DNA, ultrasound, and amniocyte karyotypes include 6 cases of inconsistent results plus 8 cases of abnormal NIPT results confirmed by karyotyping. These positive abnormal frequencies of circulating trophoblastic DNAs ~15% (15 of 103) are attributed to the enrichment of positive testing trophoblastic cases referred to Maternal Fetal Medicine specialists. Our last case correctly reported monosomy X in a mosaic placenta, but found a different major chromosome abnormality in a phenotypically normal newborn predicting high risk of major unanticipated difficulties at reproductive age. Presented cases highlight our attention to (1) the abnormal subsequent ultrasound following a normal circulating trophoblastic DNA (cfDNA) result on a trisomy 18 fetus, (2) the substantial proportion of abnormal mosaic placental karyotypes among all abnormal cfDNA results that may or may not be identified in the fetus, (3) the correct fetal information in advance of patient care even when the patient elects to carry an abnormal fetus, and (4) *one false positive cfDNA result followed by amniocentesis that resulted in complications necessitating extended maternal hospitalization.* Together these cases emphasize the significance of pretest counseling, following up NIPT results with targeted ultrasound and amniocentesis, and karyotyping the placenta and cord blood given inconsistent or mosaic results.

## Endnotes

^a^Fetal DNA circulating in maternal blood originating in the placenta is referenced as the target of Noninvasive Prenatal Testing (NIPT) in the remainder of this study.

^b^Licensed tests initially offered commercially, 2013:

A. The *Harmony Prenatal Test™* (Ariosa Diagnostics, San Jose, California), employs directed analysis of cell-free DNA fragments. This test selected regions (DANSR™) with a proprietary algorithm, FORTE™, to selectively analyze cell-free DNA in maternal blood for trisomy 21, 18 and 13.
B. The *Verifi™ Prenatal Test* (Verinata Health Inc., Redwood City, CA), utilizes massively parallel DNA sequencing to identify an increased representation of chromosomes 21, 18, and13. Verinata Health Inc also offers testing for Monosomy X (Turner Syndrome) as an additional option to the verifi(TM) prenatal test;
C. *MaterniT21™ Plus Test* (Sequenom Center for Molecular Medicine, Grand Rapids, MI), utilizes massively parallel DNA sequencing to identify trisomy of chromosomes 21, 18, 13, 16, and 22; 22q deletion syndrome (DiGeorge), 5p (Cri-du-chat syndrome), 15q (Prader-Willi/Angelman syndromes), 1p36 deletion syndrome.
D. Natera, Inc. 201 Industrial Road, Suite 410, San Carlos, CA 94070, (650) 249-9090] provides the Panorama prenatal test based upon single nucleotide polymorphisms.

^c^The 4 CVS samples were further analyzed by FISH for chromosome 21 and chromosome 13 copy number. Following collagenase digestion of dissected villous cells, hypotonic, and immediate fixation, FISH analysis found 50 of 50 cells with two copies of the chromosome 13q probe but 32% of these nuclei with three copies of the 21q probe [AneuVysion, Abbot]. The other three biopsied CVS samples had 68, 40, and 80% trisomy 21 nuclei among 25 scored cells each while all nuclei had two copies of the control chromosome 13 locus.

## Additional file

Additional file 1. Typical optimized NIPT protocols by Akron Children’s Hospital MFM clinic.

## Abbreviations

FACS: fluorescence activated cell sorter
cfDNA: circulating fetal DNA in maternal plasma more accurately circulating placental DNA
IUGR: intrauterine growth restriction
SGA: small for gestational age
NIPT: noninvasive prenatal testing
MPSS: massively parallel shotgun sequencing
