# Supplementary material for: Discordant circulating fetal DNA and subsequent cytogenetics reveal false negative, placental mosaic, and fetal mosaic cfDNA genotypes
Source: J Transl Med. 2015 Aug 11;13:260. doi: 10.1186/s12967-015-0569-y (PMC4531495; doi:10.1186/s12967-015-0569-y)
Supplement: Additional file 1. — Typical optimized NIPT protocols by Akron Children’s Hospital MFM clinic. [file 12967_2015_569_MOESM1_ESM.docx]

NORTHEAST OHIO NIPT PROTOCOL

Family history and/or

Parental Screen

1. Positive (1A)

Amniocentesis or CVS Molecular and/or Karyotype

Negative

Offer Amniocentesis or CVS Karyotype with possible other tests

First trimester ultrasound

1. Abnormal (2A)

Abnormal

Normal

Offer Amniocentesis and Karyotype

(3) (3A)

Offer NIPT (cfDNA)

Offer Amniocentesis and Karyotype

If amniocytes normal, Karyotype placenta when obtained.

If placenta abnormal, Karyotype blood when available

Normal Abnormal

20 Week Anatomy Ultrasound

(4) (4A**)**

Abnormal

NORMAL

(5) (5A)

Offer Amniocentesis and Karyotype

Table 2. Northeast Ohio NIPT Test Protocol.

Left column reflects entirely normal results with NIPT screening. (1-5)

1. Family history of a cytogenetic, metabolic, or molecular genetic disease or positive parental screening for a genetic disease will be offered amniocentesis for molecular testing, karyotyping, or both.
2. An abnormal first trimester ultrasound is followed regularly by amniocentesis provides a sample for amniocyte cultures and fluid that can be karyotyped and tested for gene mutations and copy number changes along with metabolites.
3. Depending upon maternal age, pregnant patients with no family history of a genetic disease and a normal fetal ultrasound can be offered NIPT and/or amniocentesis. This is the primary decision made by the patient to decide whether to elect maternal blood screening to avoid fetal risk of an invasive fetal sampling procedure or to follow up with a test that will give conclusive results. A normal result will add assurance that about 74% of detectable chromosome abnormalities are not carried by the fetus. An abnormal result will substantially increase the risk the fetus has a chromosome abnormality but will not confirm this conclusion without completing a follow up amniocentesis.(4A) An abnormal amniocentesis karyotype will confirm the result. A normal amniocentesis result will confirm this fetus does not carry the NIPT reported abnormal karyotype in fetal amniocytes which include fetal skin, the buccal cavity, and developing lungs bathed by amniotic fluid.
4. A normal NIPT result is followed by a 20 week anatomic ultrasound to further confirm the normal NIPT result. This completes NIPT testing for an otherwise normal pregnancy when an ultrasound reveals a visible as well as any other appropriate test to characterize the source of the testable abnormalities.
